# Metagenome-assembled genomes from the temperate forest phyllosphere in Eastern Canada

**DOI:** 10.1099/acmi.0.001189.v3

**Published:** 2026-07-02

**Authors:** David A.N. Ross, Jocelyn Lauzon, Vladimir Makarenkov, Steven W. Kembel

**Affiliations:** 1 Département d’informatique, Université du Québec à Montréal, Montréal, Québec, Canada; 2 Département des sciences biologiques, Université du Québec à Montréal, Montréal, Québec, Canada

**Keywords:** *Beijerinckiaceae*, metagenomics, phyllosphere, *Pseudomonadota*

## Abstract

The phyllosphere is host to diverse microbial communities surviving in dynamic environmental conditions and which form important relationships with their hosts. Here, we constructed metagenome-assembled genomes (MAGs) from 25 temperate forest phyllosphere samples collected in Eastern Canada. We found 423 dereplicated MAGs with completeness ≥50% and contamination ≤10%, using a combination of co-assembly strategies. The MAGs were predominantly classified into the bacterial phyla *Pseudomonadota* (*n*=197), *Actinomycetota* (*n*=88) and *Acidobacteriota* (*n*=50) and included two archaeal MAGs in the phylum *Thermoproteota*. These genomes can help to improve reference database entries of phyllosphere-affiliated microbes, increasing our understanding of phyllosphere microbial phylogenomic and community dynamics and the ecological roles of phyllosphere microbiomes.

**Impact Statement**

The goal of this research was to identify known and novel microbial genomes from phyllosphere metagenomic samples. We identified 423 genomes comprising known and unknown species, whose taxonomic identities broadly agreed with known phyllosphere community composition. These genomes will be useful to expand our understanding of the genomic repertoire of phyllosphere microbes and to improve the ability to taxonomically and functionally identify microbes living on leaf surfaces by improving reference databases.

## Data Summary

The raw data and metagenome-assembled genomes generated in this study are available through the European Nucleotide Archive (ENA) and can be found under the project accession PRJEB93989. Sequencing reads are available under accession numbers ERS27090401-25. MAGs are available under accession numbers ERS27163163-585 (as listed in Table S1).

## Introduction

The phyllosphere, the aboveground organs of plants including leaves, is a highly dynamic environment for microbial life [1,2]. Bacteria thriving on leaf surfaces are exposed to high UV radiation, rapidly changing temperature and humidity and often drastic seasonal variation [3], including yearly leaf senescence in seasonal forests. Furthermore, host plants secrete a variety of chemical compounds that can act as antimicrobials [4] or as carbon sources [5]. Leaf bacteria play key ecological roles in host health, ranging from pathogens [6] to growth promoters [7], as well as in ecosystem functioning, notably through nutrient cycling [8,9] and diversity-mediated ecosystem productivity enhancement [10].

In this study, we report 421 bacterial and 2 archaeal metagenome-assembled genomes (MAGs) recovered from the temperate forest phyllosphere in Eastern Canada. Samples were collected as part of a study [11], investigating tree host species adaptations of *Methylobacterium* – a facultative methylotroph bacterial genus ubiquitous in the phyllosphere known to stimulate plant growth [5,12,14]. The reported genomes will provide an important contribution to our understanding of the microbial communities of the phyllosphere ecosystem and to the importance of host–microbe interactions in plant and microbial ecology.

## Description of the dataset

Leaf samples were collected from the subcanopy (~1.5–5 m height, depending on tree height and leaf availability) of five host plant species growing at the Station de biologie des Laurentides, located in Saint-Hippolyte, Québec, Canada (45.99° N 73.99° W) in July 2023. One sample was taken from five individuals each of the coniferous tree species *Abies balsamea* and *Thuya occidentalis*, the deciduous tree species *Acer saccharum* and *Fagus grandifolia* and the deciduous shrub species *Corylus cornuta*. Samples were transferred immediately to a sterile collection bag, for a total of 25 samples. The leaf surface microbial community of each sample was isolated using a phosphate buffer wash following the protocol described by Leducq *et al*. [13]. Metagenomic DNA was extracted using the DNeasy PowerSoil Pro Kit (QIAGEN), and libraries were prepared with the NEBNext Ultra II FS DNA Library Prep Kit for Illumina (New England Biolabs) at the Center of Excellence in Research on Orphan Diseases-Fondation Courtois (CERMO-FC) Genomics Platform at the University of Quebec in Montreal (Québec, Canada). Paired-end metagenomic shotgun sequencing was performed using the Illumina NovaSeq 6000 S4 flow cell at the Centre de recherche CHU de Québec (Laval University, Québec, Canada). A total of 2.13B paired-end reads were obtained (323.31 Gb), ranging from 54.3M to 123.6M reads per sample (8.25–18.78 Gb) (see Table S2, available in the online Supplementary Material).

MAGs were generated using a modified metaWRAP [15] pipeline. Reads were trimmed with Trim Galore v0.5.0 [16], host reads were removed using BMTagger v1.1.0 [17] and quality reports were produced with FastQC v0.11.8 [18]. A host species reference genome was available only for *A. saccharum*; *Corylus avellana* was used as a reference genome for *C. cornuta* and *Fagus sylvatica* for *F. grandifolia*. Host reads were not removed for *A. balsamea* nor *T. occidentalis*. Filtered sequences were assembled into contigs using two approaches to sample assembly. First, the five samples per host species were concatenated and co-assembled using metaSPAdes v3.15.4 (adjusted parameter: --only-assembler) [19]. Second, a full co-assembly of all 25 samples was performed using MEGAHIT v1.2.9 (adjusted parameter: --presets, meta-large) [20]. Each assembly was independently binned using MaxBin 2.0 v2.2.6 [21], MetaBAT 2 v2.12.1 [22] and CONCOCT v1.0.0 [23], which were refined with metaWRAP’s bin_refinement module, using CheckM v1.2.1 [24] with a cutoff of ≥50% completeness and ≤10% contamination. MetaWRAP’s bin_reassembly module was used to improve bin quality where possible. Due to computational limits, MaxBin 2.0 and bin_reassembly were not applied to the full co-assembly. All MAGs were then dereplicated with dRep v3.5.0 (adjusted parameter: --sa 0.99, -comp 50) [25] to produce 423 unique MAGs (≥99% average nucleotide identity). Genome coverage was calculated using CoverM v0.7.0 [26].

To investigate the effect of sample co-assembly on MAG recovery, we additionally generated a set of dereplicated MAGs from only the per-host species assemblies and compared them with our final set of 423 MAGs (which includes the full co-assembly of all 25 samples). The full co-assembly generated 120 unique genomes, of which 73 were retained in the 423 MAGs reported here, 60 of which were unique to the full co-assembly output.

Taxonomic classification was performed with GTDB-Tk v2.3.2 [27] using the Genome Taxonomy Database (GTDB) v08 release R214 [28]. Of the 423 MAGs (Table 1, Fig. S2), 421 were classified as *Bacteria*, predominantly in the phyla *Pseudomonadota* (*n*=197), *Actinomycetota* (*n*=88) and *Acidobacteriota* (*n*=50). The two *Archaea* MAGs were both classified to the undescribed class *JANJXX01*, in the phylum *Thermoproteota*.

**Table 1.** Summary of 423 MAGs recovered from temperate forest phyllosphere samples in Eastern Canada, grouped by family

MAGs identified to family	n	Completeness	Contamination	Quality score	GC	N50	Size (Mb)
Archaea							
Thermoproteota							
Unidentified*	2	51.5–53.1	7.1–7.9	13.6–16.1	0.47–0.55	3,589–9,566	2.0–7.4
Bacteria							
*Pseudomonadota*							
*Beijerinckiaceae*	51	50.3–91.7	0.0–9.5	14.8–84.4	0.62–0.73	2,600–15,990	2.1–5.4
*Acetobacteraceae*	40	50.0–95.4	0.0–9.6	16.4–89.9	0.57–0.72	2,954–23,816	2.2–6.4
*Sphingomonadaceae*	20	50.6–75.7	0.0–5.2	26.9–62.0	0.64–0.71	2,813–8,903	2.0–3.4
*Rickettsiaceae*	18	55.5–99.5	0.6–9.3	33.8–95.2	0.30–0.34	3,961–376,014	0.9–1.6
*Burkholderiaceae*_B	10	51.4–96.2	0.0–7.5	28.3–89.3	0.69–0.72	2,855–15,788	2.4–4.8
*Burkholderiaceae*	7	53.8–87.5	1.4–6.4	23.7–80.4	0.61–0.71	2,107–17,513	2.3–8.4
*Enterobacteriaceae*	6	75.8–100.0	0.0–2.9	70.5–100.0	0.19–0.56	3,823–120,386	0.6–5.5
*Pseudomonadaceae*	6	50.9–98.6	0.0–4.4	28.9–94.7	0.55–0.64	4,529–72,272	4.4–6.5
*Caedimonadaceae*	5	57.2–80.9	0.0–8.4	23.0–80.9	0.33–0.42	3,852–113,991	1.2–1.5
*Caulobacteraceae*	5	52.2–77.5	1.6–9.3	5.6–69.3	0.66–0.72	2,413–11,792	1.7–5.7
*Arcanobacteraceae*	3	70.6–98.9	0.5–4.9	46.4–96.2	0.27–0.28	3,761–123,289	1.1–1.6
*Midichloriaceae*	3	93.4–100.0	0.5–1.1	87.9–97.3	0.31–0.33	15,409–184,321	1.1–1.4
UBA9215	2	59.1–62.8	0.1–1.1	53.7–62.3	0.40–0.40	4,483–6,108	0.8–0.9
*Legionellaceae*	2	82.1–96.8	1.3–3.3	65.5–90.1	0.36–0.37	19,583–49,189	2.0–2.7
JAJYDF01	1	90.6	0.0	90.6	0.38	23,790	1.3
CAIULA01	1	90.3	0.0	90.3	0.36	11,831	1.5
*Rhizobiaceae*	1	65.6	2.9	51.0	0.67	3,298	3.0
*Diplorickettsiaceae*	1	93.0	0.0	93.0	0.37	44,5110	1.5
Unidentified*	15	56.5–96.8	0.0–9.3	30.4–96.8	0.32–0.41	3,538–399,529	0.8–1.8
*Actinomycetota*							
*Microbacteriaceae*	26	50.0–87.5	0.0–7.9	17.6–84.7	0.66–0.74	2,650–16,955	1.5–3.8
*Jatrophihabitantaceae*	21	50.8–92.3	0.0–8.2	18.5–86.5	0.66–0.72	2,984–31,659	2.3–4.9
*Propionibacteriaceae*	9	52.0–90.7	0.0–9.6	25.8–82.7	0.69–0.74	3,206–13,071	2.7–4.2
*Pseudonocardiaceae*	5	57.8–83.9	1.1–2.9	45.8–77.8	0.73–0.75	2,635–11,301	3.0–4.8
*Mycobacteriaceae*	4	72.4–94.8	0.9–2.1	62.2–88.2	0.68–0.69	3,279–30,381	4.5–5.6
*Nakamurellaceae*	3	65.0–94.4	0.8–1.7	57.8–89.1	0.69–0.69	3,651–21,808	3.8–4.4
70-9	3	50.6–60.8	1.3–6.9	26.4–44.1	0.69–0.70	2,562–8,570	3.1–3.4
*Nocardioidaceae*	3	50.1–84.8	0.8–4.3	28.5–77.0	0.70–0.73	3,022–8,463	2.8–3.7
*Kineosporiaceae*	2	52.1–55.1	5.2–5.8	26.1–26.2	0.73–0.73	44,52–7,269	5.1–5.5
JACCYY01	2	77.3–83.5	1.8–2.6	68.3–70.4	0.72–0.74	4,713–13,069	4.0–4.1
*Frankiaceae*	2	72.2–75.8	3.7–8.4	30.4–57.1	0.71–0.72	4,821–8,114	3.4–3.5
*Kineococcaceae*	2	65.8–81.7	3.4–4.1	45.3–64.8	0.72–0.72	4,735–5,403	4.5–4.7
*Quadrisphaeraceae*	2	78.9–84.5	2.0–8.3	37.7–74.6	0.75–0.75	5,931–9,304	3.6–4.0
CAJCIY01	1	74.5	0.0	74.5	0.70	14,179	2.4
Unidentified*	3	55.2–65.1	0.5–9.0	10.4–62.4	0.73–0.74	2,344–10,803	3.5–4.3
*Acidobacteriota*							
*Acidobacteriaceae*	50	51.1–92.2	0.0–9.5	7.2–85.3	0.58–0.65	2,273–59,374	2.0–5.1
*Bacteroidota*							
*Spirosomaceae*	16	53.5–99.4	0.0–5.9	30.3–97.0	0.46–0.56	2,588–273,973	3.1–7.0
*Chitinophagaceae*	11	53.7–91.3	0.0–6.7	25.4–87.8	0.36–0.51	1,976–18,908	2.2–4.9
*Sphingobacteriaceae*	9	52.3–93.5	0.6–6.4	28.3–89.5	0.37–0.45	2,522–37,747	2.6–4.7
*Hymenobacteraceae*	2	51.8–66.4	0.0–8.8	7.9–66.4	0.62–0.65	3,112–5,690	4.1–5.6
*Weeksellaceae*	1	55.4	1.0	50.5	0.34	3,510	1.0
*Bdellovibrionota*							
UBA6776	8	50.9–92.2	1.1–6.1	33.3–78.0	0.43–0.62	3,095–17,108	2.1–3.9
JAMPXM01	6	53.5–94.1	0.0–9.0	23.9–93.7	0.50–0.54	2,868–44,431	2.7–4.9
CAIPTA01	3	58.8–76.5	0.0–3.5	41.2–67.7	0.54–0.55	2,958–6,716	1.8–3.0
*Myxococcota*							
SYDY01	4	59.5–85.2	0.8–7.4	48.0–64.1	0.57–0.65	5,174–14,422	1.9–7.2
*Myxococcaceae*	3	54.9–79.9	3.9–8.8	11.0–60.3	0.70–0.70	3,662–4,103	7.0–7.3
*Polyangiaceae*	2	52.4–53.3	0.4–1.5	45.8–50.1	0.69–0.69	2,571–2,735	3.3–3.8
JALHOP01	2	65.9–86.5	1.7–2.5	57.2–74.2	0.60–0.66	3,280–6,685	4.6–6.4
JAEUJZ01	1	80.5	5.5	53.1	0.67	5,963	8.0
*Armatimonadota*							
*Abditibacteriaceae*	3	55.0–91.7	0.1–6.1	24.4–91.2	0.56–0.57	2,421–65,395	4.9–6.6
*Capsulimonadaceae*	1	86.8	1.2	80.6	0.62	20,655	4.0
*Chlamydiota*							
FEN-1388	1	87.0	0.7	83.6	0.36	53,413	1.6
JABDDJ01	1	98.6	2.0	88.5	0.43	94,882	2.1
*Simkaniaceae*	1	57.2	0.0	57.0	0.39	2,821	0.9
*Parachlamydiaceae*	1	68.8	8.5	26.2	0.32	3,234	2.2
*Deinococcota*							
*Deinococcaceae*	3	50.3–82.2	1.7–6.6	34.5–66.0	0.66–0.68	2,960–5,191	2.9–3.8
*Dependentiae*							
Unidentified*	3	76.0–81.4	0.0–0.0	76.0–81.4	0.35–0.35	45,180–780,614	0.8–0.9
*Patescibacteria*							
UBA4665	1	65.4	1.0	60.3	0.49	4,378	1.3
Unidentified*	1	53.4	0.5	50.8	0.54	4,594	0.6
*Planctomycetota*							
*Isosphaeraceae*	1	84.8	1.3	78.3	0.66	6,150	6.9
*Verrucomicrobiota*							
Unidentified*	1	63.3	0.7	60.0	0.37	7,872	1.0

Completeness, contamination, GC, N50 and size metrics were measured using CheckM v1.2.1; quality score is measured as [completeness − (5 x contamination)].

*The MAGs assigned to the given phylum but not classified to family level.

Within *Pseudomonadota*, the MAGs were predominantly (*n*=164) classified in *Alphaproteobacteria*, of which *Rhizobiales* (*n*=52), *Acetobacterales* (*n*=40) and *Rickettsiales* (*n*=27) were the most common orders. *Rhizobiales* were almost entirely classified within *Beijerinckiaceae* (*n*=51), comprising the described genera *Lichenihabitans* (*n*=17), *Methylobacterium* (*n*=12) and *Enterovirga* (*n*=1); the undescribed genera *RH-AL1* (*n*=13) and *JAJXWB01* (*n*=5) and 3 unclassified genera. The order *Acetobacterales* contained only the family *Acetobacteraceae* (*n*=40), where the dominant genera were *Rhodopila* (*n*=9) and the unclassified genus *CAHJXG01* (*n*=13). *Rickettsiaceae* (*n*=18) was the dominant family within *Rickettsiales* and contained only the undescribed genera *JAKONF01* (*n*=9) and *GCF-002259525* (*n*=3), as well as 6 unclassified genera.

MAGs classified as *Actinomycetota* were predominantly from the class *Actinomycetia* (*n*=85), which was comprised mostly of the orders *Mycobacteriales* (*n*=36) and *Actinomycetales* (*n*=33), dominated by the families *Jatrophihabitantaceae* (*n*=21) and *Microbacteriaceae* (*n*=26) respectively. MAGs in the family *Jatrophihabitantaceae* were comprised mostly of the genera *Jatrophihabitans_A* (*n*=12) and *Jatrophihabitans* (*n*=5), while the family *Microbacteriaceae* contained mostly the genera *Amnibacterium* (*n*=14), *Frondihabitans* (*n*=6) and *Subtercola* (*n*=4).

MAGs in the phylum *Acidobacteriota* were entirely classified in the class *Terriglobia* (*n*=50), the order *Terriglobales* (*n*=50), the family *Acidobacteriaceae* (*n*=50) and predominantly in the genus *Terriglobus* (*n*=23).

Only the MAG ‘acsa_bin.29.orig’ (ERS27163290) met the MIMAG [29] high-quality draft standard. It had a completeness of 98.9%, contamination of 0.549%, was 1.58 Mb, containing 15 contigs, with an N_50_ value of 123,289 bp and guanine-cytosine (GC) content of 27.4%. Fifty-four of the medium-quality genomes met the high-quality completeness and contamination standards, with values from 90.7–100.0% and 0.0–4.4%, respectively. These genomes ranged from 0.64 to 7.03 Mb, containing 7–572 contigs, with N_50_ values from 8,739 to 445,110 bp and GC content between 25.8 and 72.9%. The remaining 368 medium-quality genomes ranged from 0.60 to 8.44 Mb, containing 1–1,283 contigs, with N_50_ values from 1,976 to 780,614 bp and GC content between 18.8 and 75.4%. Bin completeness ranged from 50.1 to 92.3% and contamination from 0.0 to 9.6%.

## Future outlook

Recovery of MAGs has become a vital tool in studying environmental microbial community dynamics. Here, we report 423 MAGs from the temperate forest phyllosphere, comprising 405 unidentified species of bacteria from 13 phyla and 2 unidentified species of archaea from a single phylum. *Beijerinckiaceae* is largely ubiquitous in the phyllosphere [30] and indeed was the most common family recovered from our samples, including 6 unidentified species from the genus *Methylobacterium* and 17 from the genus *Lichenihabitans*. These data will benefit future studies of phyllosphere microbial phylogenomic and community dynamics and help increase our understanding of the influence of host–microbe interactions on plant physiology and terrestrial ecosystem functioning. The novel MAGs identified in this study will also be useful to improve our ability to taxonomically and functionally annotate microbial sequences from leaf surfaces, which are currently poorly represented in reference databases.

Our comparison of per-host-species versus all-samples co-assembly suggested that co-assembly of all samples can complement per-sample (or grouped-sample) assembly output by identifying more rare genomes (Fig. S1); however, this approach had its limits. Compared with per-host-species assemblies, for full co-assembly, we observed fewer MAGs recovered from the overall most predominant bacterial families and a significant increase in the contamination values of MAGs (Multivariate analysis of variance: Pillais’ trace=0.12, F_(10, 1014)_ = 6.28, *P*<0.001; ANOVA: F_(5, 507)_ = 11.21, *P*<0.001; Figs S1 and S2), suggesting a trade-off between recovery of common versus rare species. As expected, when the concatenated read files were mapped back onto the final MAGs, the MAGs generated from each host-species-specific co-assembly showed the highest fraction covered and coverage depth (Fig. S3A–E). Similarly, as the MAGs generated from the full co-assembly include reads from all host species, we would expect their mapping results to resemble those of the four non-corresponding host-species-specific assemblies. The fully concatenated reads mapped onto the MAGs (Fig. S3F) showed a cumulative effect of the five previous plots. We recommend that, when possible, these approaches be used in tandem to maximize MAG recovery.

## Supplementary material

10.1099/acmi.0.001189.v3 Supplementary Material 1.

## Abbreviations

GC: guanine-cytosine
GTDB: genome taxonomy database
MAG: metagenome-assembled genome
