# Supplementary material for: Metagenome-assembled genomes from the temperate forest phyllosphere in Eastern Canada
Source: Access Microbiol. 2026 Jul 2;8(7):001189.v3. doi: 10.1099/acmi.0.001189.v3 (PMC13327642; doi:10.1099/acmi.0.001189.v3)

SUPPLEMENTARY TABLES

Table S1: Details and NCBI accessions of 423 MAGs recovered from temperate forest phyllosphere samples in Eastern Canada. Completeness (comp.), contamination (cont.), GC, N50, and size metrics were measured using CheckM v1.2.1, quality score is measured as [completeness - 5*contamination]. Taxonomy was assigned with GTDB-Tk v2.3.2 using the GTDB v08 release R214.

MAG	Accession	Comp.	Cont.	Quality Score	GC	N50	Size (Mb)	Family	Species
Archaea									
thoc_bin.78.orig	ERS27163565	53.12	7.907	13.585	0.553	9566	2.011557	Thermoproteota archaeon	-
fagr_bin.35.orig	ERS27163452	51.52	7.081	16.115	0.472	3589	7.43344	Thermoproteota archaeon	-
Bacteria									
Acidobacteriota									
fagr_bin.56.strict	ERS27163473	79.07	2.209	68.025	0.606	19960	3.933912	Acidobacteriaceae	*Bryocella sp.*
acsa_bin.5.orig	ERS27163298	64.93	0.862	60.62	0.605	8393	3.082336	Acidobacteriaceae	*Bryocella sp.*
abba_bin.107.orig	ERS27163169	51.45	0.407	49.415	0.621	5969	1.997922	Acidobacteriaceae	*Bryocella sp.*
thoc_bin.91.orig	ERS27163579	83.77	3.328	67.13	0.619	5670	3.548172	Acidobacteriaceae	*CAHJWL01 sp.*
abba_bin.67.orig	ERS27163241	75.35	1.025	70.225	0.61	10366	3.699239	Acidobacteriaceae	*CAHJWL01 sp.*
abba_bin.93.strict	ERS27163266	75.22	1.282	68.81	0.628	19082	3.227776	Acidobacteriaceae	*CAHJWL01 sp.*
abba_bin.11.orig	ERS27163182	66.76	1.549	59.015	0.619	5607	3.530086	Acidobacteriaceae	*CAHJWL01 sp.*
thoc_bin.76.orig	ERS27163563	62.9	1.709	54.355	0.611	7974	2.767031	Acidobacteriaceae	*CAHJWL01 sp.*
coco_bin.26.orig	ERS27163393	56.68	2.26	45.38	0.609	3709	2.851337	Acidobacteriaceae	*CAHJWL01 sp.*
thoc_bin.22.orig	ERS27163508	54.29	6.196	23.31	0.643	3915	2.695612	Acidobacteriaceae	*CAHJWL01 sp.*
fagr_bin.43.strict	ERS27163460	62.98	2.831	48.825	0.617	7213	2.866855	Acidobacteriaceae	*CAIQPK01 sp.*
all_bin.93	ERS27163370	65.51	1.724	56.89	0.629	44163	3.154007	Acidobacteriaceae	*EB88 sp.*
fagr_bin.32.orig	ERS27163449	88.58	1.933	78.915	0.621	13950	3.389965	Acidobacteriaceae	*Granulicella_C sp.*
abba_bin.81.orig	ERS27163256	73.24	1.709	64.695	0.612	4188	2.741111	Acidobacteriaceae	*Granulicella_C sp.*
abba_bin.2.strict	ERS27163202	70.63	1.24	64.43	0.626	11548	3.241369	Acidobacteriaceae	*Granulicella_C sp.*
all_bin.100	ERS27163303	68.96	9.482	21.55	0.621	10184	2.8195	Acidobacteriaceae	*Granulicella_C sp.*
fagr_bin.10.orig	ERS27163430	61.6	2.35	49.85	0.638	4184	2.693139	Acidobacteriaceae	*Granulicella_C sp.*
fagr_bin.22.strict	ERS27163442	55.48	3.448	38.24	0.628	7256	2.591406	Acidobacteriaceae	*Granulicella_C sp.*
abba_bin.99.strict	ERS27163272	55.11	4.653	31.845	0.651	4748	2.750805	Acidobacteriaceae	*Granulicella_C sp.*

fagr_bin.65.orig	ERS27163481	53.44	9.247	7.205	0.632	2273	3.089465	Acidobacteriaceae	*Granulicella_C sp.*
fagr_bin.67.orig	ERS27163483	84.16	0.862	79.85	0.621	19420	3.39637	Acidobacteriaceae	*Terriglobus sp.*
thoc_bin.44.strict	ERS27163531	83.84	4.148	63.1	0.632	7457	3.708216	Acidobacteriaceae	*Terriglobus sp.*
abba_bin.122.strict	ERS27163185	82.78	1.778	73.89	0.63	59374	3.893967	Acidobacteriaceae	*Terriglobus sp.*
thoc_bin.40.strict	ERS27163527	77.88	0.915	73.305	0.621	6164	3.354668	Acidobacteriaceae	*Terriglobus sp.*
abba_bin.69.orig	ERS27163243	77.59	1.346	70.86	0.583	6749	3.511497	Acidobacteriaceae	*Terriglobus sp.*
thoc_bin.6.orig	ERS27163557	68.59	7.758	29.8	0.611	3861	3.117629	Acidobacteriaceae	*Terriglobus sp.*
abba_bin.27.strict	ERS27163199	67.89	0	67.89	0.605	25321	3.07674	Acidobacteriaceae	*Terriglobus sp.*
fagr_bin.59.orig	ERS27163476	67.72	0.431	65.565	0.601	6421	3.018996	Acidobacteriaceae	*Terriglobus sp.*
abba_bin.5.strict	ERS27163234	64.45	2.586	51.52	0.605	11594	2.9357	Acidobacteriaceae	*Terriglobus sp.*
fagr_bin.45.strict	ERS27163462	63.94	0	63.94	0.624	7319	2.598056	Acidobacteriaceae	*Terriglobus sp.*
fagr_bin.38.strict	ERS27163455	62.99	3.057	47.705	0.626	15482	3.20697	Acidobacteriaceae	*Terriglobus sp.*
all_bin.24	ERS27163323	60.91	8.62	17.81	0.601	7581	3.487847	Acidobacteriaceae	*Terriglobus sp.*
fagr_bin.17.orig	ERS27163436	60.65	2.586	47.72	0.636	7242	2.59066	Acidobacteriaceae	*Terriglobus sp.*
abba_bin.111.strict	ERS27163173	58.94	2.155	48.165	0.607	9623	4.55716	Acidobacteriaceae	*Terriglobus sp.*
coco_bin.47.strict	ERS27163415	54.17	2.873	39.805	0.62	9168	2.801438	Acidobacteriaceae	*Terriglobus sp.*
acsa_bin.16.orig	ERS27163279	53.2	1.724	44.58	0.618	4692	2.320621	Acidobacteriaceae	*Terriglobus sp.*
acsa_bin.15.orig	ERS27163278	52.58	4.31	31.03	0.6	2616	2.29738	Acidobacteriaceae	*Terriglobus sp.*
thoc_bin.57.permissive	ERS27163545	52.58	1.724	43.96	0.624	5268	2.478452	Acidobacteriaceae	*Terriglobus sp.*
fagr_bin.13.orig	ERS27163432	52.3	0.313	50.735	0.63	3605	2.875589	Acidobacteriaceae	*Terriglobus sp.*
coco_bin.13.orig	ERS27163379	52.1	2.065	41.775	0.619	2920	2.05419	Acidobacteriaceae	*Terriglobus sp.*
abba_bin.95.strict	ERS27163268	51.98	5.603	23.965	0.618	3943	2.815844	Acidobacteriaceae	*Terriglobus sp.*
fagr_bin.4.orig	ERS27163466	51.51	0.215	50.435	0.619	5957	2.284003	Acidobacteriaceae	*Terriglobus sp.*
coco_bin.49.orig	ERS27163417	51.13	0	51.13	0.632	5736	2.216287	Acidobacteriaceae	*Terriglobus sp.*
abba_bin.26.orig	ERS27163198	92.16	1.364	85.34	0.619	16239	5.071515	Acidobacteriaceae	*Tous-C9LFEB sp.*
fagr_bin.24.orig	ERS27163444	72.38	5.009	47.335	0.605	7292	3.078889	Acidobacteriaceae	*Tous-C9LFEB sp.*
thoc_bin.41.orig	ERS27163528	64.65	1.724	56.03	0.617	3408	2.781589	Acidobacteriaceae	*Tous-C9LFEB sp.*
abba_bin.71.strict	ERS27163246	63.71	0	63.71	0.626	13066	2.789721	Acidobacteriaceae	*Tous-C9LFEB sp.*
all_bin.96	ERS27163372	55.78	3.836	36.6	0.632	6101	2.173171	Acidobacteriaceae	*Tous-C9LFEB sp.*
all_bin.51	ERS27163342	52.58	3.793	33.615	0.625	7915	3.075491	Acidobacteriaceae	*Tous-C9LFEB sp.*
abba_bin.118.orig	ERS27163180	78.67	0.862	74.36	0.616	8080	3.769881	Acidobacteriaceae	-

Actinomycetota									
coco_bin.17.orig	ERS27163383	81.7	3.378	64.81	0.718	4735	4.650721	Kineococcaceae	*Kineococcus sp.*
all_bin.27	ERS27163326	65.83	4.099	45.335	0.716	5403	4.46032	Kineococcaceae	*Kineococcus sp.*
all_bin.30	ERS27163328	55.09	5.799	26.095	0.727	7269	5.522138	Kineosporiaceae	-
abba_bin.44.orig	ERS27163218	52.06	5.172	26.2	0.728	4452	5.111219	Kineosporiaceae	-
coco_bin.3.orig	ERS27163407	81.44	1.01	76.39	0.74	14144	2.329989	Microbacteriaceae	*Amnibacterium sp.*
abba_bin.57.orig	ERS27163231	61.4	7.894	21.93	0.738	2895	2.248357	Microbacteriaceae	*Amnibacterium sp.*
abba_bin.45.strict	ERS27163219	60.15	7.017	25.065	0.735	3454	2.238658	Microbacteriaceae	*Amnibacterium sp.*
abba_bin.38.strict	ERS27163211	58.31	2.194	47.34	0.723	6307	3.119343	Microbacteriaceae	*Amnibacterium sp.*
thoc_bin.54.orig	ERS27163542	54.15	1.754	45.38	0.736	3207	1.951961	Microbacteriaceae	*Amnibacterium sp.*
thoc_bin.86.orig	ERS27163573	52.77	2.631	39.615	0.738	3203	2.191492	Microbacteriaceae	*Amnibacterium sp.*
thoc_bin.100.strict	ERS27163488	52.44	0	52.44	0.736	3699	2.128435	Microbacteriaceae	*Amnibacterium sp.*
fagr_bin.39.orig	ERS27163456	52.11	1.451	44.855	0.738	2650	1.523074	Microbacteriaceae	*Amnibacterium sp.*
fagr_bin.11.orig	ERS27163431	51.75	3.411	34.695	0.724	2791	1.882975	Microbacteriaceae	*Amnibacterium sp.*
abba_bin.55.orig	ERS27163230	51.06	4.146	30.33	0.741	3731	2.292709	Microbacteriaceae	*Amnibacterium sp.*
thoc_bin.65.orig	ERS27163553	51.03	0.584	48.11	0.738	2933	1.868788	Microbacteriaceae	*Amnibacterium sp.*
abba_bin.115.orig	ERS27163177	50.87	3.508	33.33	0.734	3560	2.657803	Microbacteriaceae	*Amnibacterium sp.*
abba_bin.116.strict	ERS27163178	50.35	0	50.35	0.729	8027	2.747325	Microbacteriaceae	*Amnibacterium sp.*
thoc_bin.70.orig	ERS27163558	50.05	1.754	41.28	0.731	3487	2.580324	Microbacteriaceae	*Amnibacterium sp.*
all_bin.14	ERS27163317	84.45	5.568	56.61	0.708	16955	3.785327	Microbacteriaceae	*Curtobacterium pusillum_A*
coco_bin.14.orig	ERS27163380	87.49	0.563	84.675	0.709	6078	2.989826	Microbacteriaceae	*Frigoribacterium sp.*
coco_bin.55.orig	ERS27163420	71.43	0	71.43	0.689	6130	2.668199	Microbacteriaceae	*Frondihabitans sp.*
fagr_bin.21.orig	ERS27163441	66.19	1.599	58.195	0.695	3766	3.128253	Microbacteriaceae	*Frondihabitans sp.*
abba_bin.42.permissive	ERS27163216	59.74	0.701	56.235	0.696	3630	3.215698	Microbacteriaceae	*Frondihabitans sp.*
abba_bin.6.permissive	ERS27163244	58.75	1.754	49.98	0.698	4493	2.633972	Microbacteriaceae	*Frondihabitans sp.*
thoc_bin.28.orig	ERS27163513	50.14	6.505	17.615	0.7	2772	2.625133	Microbacteriaceae	*Frondihabitans sp.*
coco_bin.60.orig	ERS27163425	60.52	0	60.52	0.706	6102	2.962302	Microbacteriaceae	*Frondihabitans sp003752365*
abba_bin.15.strict	ERS27163188	62.32	2.948	47.58	0.686	4511	3.135366	Microbacteriaceae	*Subtercola sp.*
all_bin.40	ERS27163334	58.67	6.14	27.97	0.662	5112	3.452535	Microbacteriaceae	*Subtercola sp.*
all_bin.64	ERS27163350	55.89	6.329	24.245	0.692	5271	2.748493	Microbacteriaceae	*Subtercola sp.*
thoc_bin.33.orig	ERS27163519	51.38	3.508	33.84	0.694	3635	2.604777	Microbacteriaceae	*Subtercola sp.*

coco_bin.34.orig	ERS27163401	84.54	1.981	74.635	0.748	9304	3.960715	Quadrisphaeraceae	*Quadrisphaera sp.*
all_bin.69	ERS27163353	78.92	8.252	37.66	0.754	5931	3.587085	Quadrisphaeraceae	*Quadrisphaera sp.*
all_bin.112	ERS27163311	83.5	2.612	70.44	0.72	13069	4.092834	JACCYY01	-
thoc_bin.96.orig	ERS27163583	77.3	1.801	68.295	0.739	4713	4.005094	JACCYY01	-
thoc_bin.39.orig	ERS27163525	74.48	0	74.48	0.703	14179	2.433745	CAJCIY01	*CAJCIY01 sp.*
thoc_bin.26.orig	ERS27163511	75.82	3.745	57.095	0.715	4821	3.488201	Frankiaceae	-
all_bin.120	ERS27163316	72.18	8.352	30.42	0.714	8114	3.380822	Frankiaceae	-
abba_bin.24.orig	ERS27163196	70.31	2.155	59.535	0.707	4874	3.037702	Jatrophihabitantaceae	*Jatrophihabitans sp.*
thoc_bin.5.orig	ERS27163548	66.19	1.168	60.35	0.714	4904	2.799486	Jatrophihabitantaceae	*Jatrophihabitans sp.*
thoc_bin.13.orig	ERS27163501	61.56	8.24	20.36	0.716	4330	3.465272	Jatrophihabitantaceae	*Jatrophihabitans sp.*
abba_bin.30.strict	ERS27163203	57.45	4.739	33.755	0.685	4523	3.038985	Jatrophihabitantaceae	*Jatrophihabitans sp.*
fagr_bin.9.orig	ERS27163487	50.79	4.283	29.375	0.71	3017	2.296596	Jatrophihabitantaceae	*Jatrophihabitans sp.*
thoc_bin.27.orig	ERS27163512	92.31	1.157	86.525	0.67	31659	4.904278	Jatrophihabitantaceae	*Jatrophihabitans_A sp.*
thoc_bin.81.orig	ERS27163569	86.5	2.586	73.57	0.682	15213	4.801221	Jatrophihabitantaceae	*Jatrophihabitans_A sp.*
all_bin.57	ERS27163345	85.33	5.506	57.8	0.688	12999	3.708456	Jatrophihabitantaceae	*Jatrophihabitans_A sp.*
abba_bin.33.orig	ERS27163206	78.38	1.985	68.455	0.663	7459	4.650486	Jatrophihabitantaceae	*Jatrophihabitans_A sp.*
abba_bin.61.orig	ERS27163236	75.98	1.323	69.365	0.697	11611	3.968554	Jatrophihabitantaceae	*Jatrophihabitans_A sp.*
all_bin.53	ERS27163343	71.71	5.49	44.26	0.662	7149	4.091916	Jatrophihabitantaceae	*Jatrophihabitans_A sp.*
all_bin.50	ERS27163341	64.82	6.896	30.34	0.679	6282	3.211162	Jatrophihabitantaceae	*Jatrophihabitans_A sp.*
abba_bin.16.orig	ERS27163189	60.15	5.336	33.47	0.686	3328	3.281954	Jatrophihabitantaceae	*Jatrophihabitans_A sp.*
abba_bin.109.orig	ERS27163171	58.28	0.778	54.39	0.716	4133	2.453297	Jatrophihabitantaceae	*Jatrophihabitans_A sp.*
all_bin.73	ERS27163355	57.06	0	57.06	0.717	7642	2.828137	Jatrophihabitantaceae	*Jatrophihabitans_A sp.*
thoc_bin.7.orig	ERS27163567	55.86	0	55.86	0.683	3943	4.232858	Jatrophihabitantaceae	*Jatrophihabitans_A sp.*
thoc_bin.34.orig	ERS27163520	51.36	0	51.36	0.687	4228	2.730642	Jatrophihabitantaceae	*Jatrophihabitans_A sp.*
abba_bin.119.orig	ERS27163181	86.09	3.855	66.815	0.683	8946	4.312502	Jatrophihabitantaceae	*QHCC01 sp.*
abba_bin.92.strict	ERS27163265	77.64	2.107	67.105	0.697	16757	3.00035	Jatrophihabitantaceae	-
abba_bin.100.orig	ERS27163163	58.96	1.724	50.34	0.681	8433	3.683614	Jatrophihabitantaceae	-
abba_bin.47.orig	ERS27163221	57.26	7.758	18.47	0.684	2984	4.078003	Jatrophihabitantaceae	-
thoc_bin.32.orig	ERS27163518	94.8	1.32	88.2	0.676	18637	5.212365	Mycobacteriaceae	*Mycobacterium sp.*
thoc_bin.30.orig	ERS27163516	90.73	0.88	86.33	0.676	30381	5.573251	Mycobacteriaceae	*Mycobacterium sp.*
thoc_bin.63.orig	ERS27163551	76.14	1.731	67.485	0.691	7822	4.688955	Mycobacteriaceae	*Mycobacterium sp.*

abba_bin.104.orig	ERS27163167	72.45	2.058	62.16	0.69	3279	4.511001	Mycobacteriaceae	*Mycobacterium sp.*
all_bin.106	ERS27163306	94.37	1.655	86.095	0.692	19722	4.137866	Nakamurellaceae	*Nakamurella sp.*
abba_bin.19.orig	ERS27163192	93.17	0.821	89.065	0.692	21808	4.422996	Nakamurellaceae	*Nakamurella sp.*
thoc_bin.31.orig	ERS27163517	64.98	1.441	57.775	0.688	3651	3.753767	Nakamurellaceae	*Nakamurella sp.*
coco_bin.4.orig	ERS27163418	83.88	1.211	77.825	0.745	11301	4.75893	Pseudonocardiaceae	*Actinomycetospora sp.*
abba_bin.48.orig	ERS27163222	78.74	1.911	69.185	0.747	4213	4.242775	Pseudonocardiaceae	*Actinomycetospora sp.*
thoc_bin.36.orig	ERS27163522	70.02	1.063	64.705	0.747	3712	3.633661	Pseudonocardiaceae	*Actinomycetospora sp.*
fagr_bin.40.orig	ERS27163458	60.53	2.938	45.84	0.742	2635	2.991102	Pseudonocardiaceae	*Actinomycetospora sp.*
abba_bin.80.orig	ERS27163255	57.81	1.724	49.19	0.732	2857	3.139581	Pseudonocardiaceae	*Actinomycetospora sp.*
thoc_bin.24.orig	ERS27163509	84.75	1.542	77.04	0.713	8463	3.651278	Nocardioidaceae	*Nocardioides sp.*
thoc_bin.4.orig	ERS27163537	55.71	0.777	51.825	0.728	3022	3.096408	Nocardioidaceae	*Nocardioides sp.*
coco_bin.5.orig	ERS27163424	50.08	4.317	28.495	0.703	6153	2.847413	Nocardioidaceae	-
abba_bin.34.orig	ERS27163207	90.72	1.597	82.735	0.729	13071	3.502517	Propionibacteriaceae	*Friedmanniella sp.*
abba_bin.98.strict	ERS27163271	66	4.058	45.71	0.731	7316	3.586266	Propionibacteriaceae	*Friedmanniella sp.*
thoc_bin.98.orig	ERS27163584	60.61	1.727	51.975	0.735	3960	2.73617	Propionibacteriaceae	*Friedmanniella sp.*
thoc_bin.56.orig	ERS27163544	56.92	0	56.92	0.728	6820	3.876585	Propionibacteriaceae	*Friedmanniella sp.*
abba_bin.59.strict	ERS27163233	68.84	2.38	56.94	0.693	7751	3.771828	Propionibacteriaceae	*Friedmanniella_A sp.*
thoc_bin.19.orig	ERS27163505	58.83	0.172	57.97	0.693	3206	2.760717	Propionibacteriaceae	*Friedmanniella_A sp.*
coco_bin.42.orig	ERS27163410	73.95	9.637	25.765	0.713	5650	4.192638	Propionibacteriaceae	*JAJTCL01 sp.*
abba_bin.77.orig	ERS27163251	79.77	4.145	59.045	0.715	8573	3.891917	Propionibacteriaceae	-
all_bin.75	ERS27163357	52.05	0.862	47.74	0.71	9691	3.845177	Propionibacteriaceae	-
all_bin.28	ERS27163327	60.84	6.896	26.36	0.693	8570	3.443065	70-9	*VAYN01 sp.*
abba_bin.94.orig	ERS27163267	56.25	4.31	34.7	0.694	4371	3.197223	70-9	*VAYN01 sp.*
thoc_bin.109.orig	ERS27163497	50.56	1.293	44.095	0.696	2562	3.133826	70-9	*VAYN01 sp.*
all_bin.45	ERS27163338	63.55	5.517	35.965	0.74	10803	4.127901	Actinomycetota bacterium	-
abba_bin.97.orig	ERS27163270	65.07	0.54	62.37	0.738	4056	3.532434	Actinomycetota bacterium	-
thoc_bin.20.orig	ERS27163507	55.22	8.965	10.395	0.73	2344	4.335583	Actinomycetota bacterium	-
Armatimonadota									
thoc_bin.95.orig	ERS27163582	91.66	0.084	91.24	0.557	65395	6.59824	Abditibacteriaceae	*Abditibacterium sp.*
fagr_bin.63.strict	ERS27163480	64.59	5.694	36.12	0.572	3001	5.002175	Abditibacteriaceae	*Abditibacterium sp.*
thoc_bin.77.strict	ERS27163564	55	6.121	24.395	0.565	2421	4.876205	Abditibacteriaceae	*Abditibacterium sp.*

thoc_bin.42.strict	ERS27163529	86.81	1.234	80.64	0.621	20655	4.045677	Capsulimonadaceae	*JAFAZD01 sp.*
Bacteroidota									
thoc_bin.61.strict	ERS27163550	91.28	1.97	81.43	0.457	12830	4.314231	Chitinophagaceae	*Ilyomonas sp.*
abba_bin.17.strict	ERS27163190	89.94	0.426	87.81	0.451	18908	4.946654	Chitinophagaceae	*Ilyomonas sp.*
thoc_bin.12.strict	ERS27163500	77.68	1.286	71.25	0.381	4743	4.032993	Chitinophagaceae	*Ilyomonas sp.*
all_bin.21	ERS27163321	62.3	6.65	29.05	0.395	3079	2.265382	Chitinophagaceae	*Ilyomonas sp.*
abba_bin.84.orig	ERS27163258	55.4	0	55.4	0.455	3012	2.165396	Chitinophagaceae	*Ilyomonas sp.*
thoc_bin.107.orig	ERS27163495	53.7	1.866	44.37	0.392	2223	2.990007	Chitinophagaceae	*Phnomibacter sp.*
abba_bin.96.orig	ERS27163269	75.6	0.519	73.005	0.512	3023	3.149114	Chitinophagaceae	*Puia sp.*
all_bin.41	ERS27163335	70.16	4.72	46.56	0.402	5578	3.556573	Chitinophagaceae	-
thoc_bin.55.strict	ERS27163543	66.91	0.985	61.985	0.444	3965	3.60425	Chitinophagaceae	-
all_bin.74	ERS27163356	59.09	1.724	50.47	0.447	5005	3.039587	Chitinophagaceae	-
thoc_bin.104.orig	ERS27163492	55.17	5.956	25.39	0.362	1976	3.668341	Chitinophagaceae	-
fagr_bin.50.orig	ERS27163467	66.37	0	66.37	0.65	5690	4.050954	Hymenobacteraceae	*Hymenobacter sp.*
thoc_bin.105.orig	ERS27163493	51.83	8.777	7.945	0.618	3112	5.573548	Hymenobacteraceae	*Hymenobacter sp.*
thoc_bin.93.strict	ERS27163581	99.39	0.483	96.975	0.46	273973	6.219641	Spirosomaceae	*Spirosoma sp.*
thoc_bin.90.orig	ERS27163578	96.7	1.488	89.26	0.563	41274	6.009074	Spirosomaceae	*Spirosoma sp.*
coco_bin.10.strict	ERS27163376	96.04	2.38	84.14	0.487	34082	7.026755	Spirosomaceae	*Spirosoma sp.*
coco_bin.58.orig	ERS27163422	95.39	2.43	83.24	0.554	20479	5.371072	Spirosomaceae	*Spirosoma sp.*
coco_bin.15.strict	ERS27163381	95.3	1.119	89.705	0.495	50039	6.629885	Spirosomaceae	*Spirosoma sp.*
abba_bin.90.strict	ERS27163263	93.61	0.313	92.045	0.469	40249	6.391446	Spirosomaceae	*Spirosoma sp.*
thoc_bin.10.strict	ERS27163498	90.76	1.398	83.77	0.481	20141	6.771349	Spirosomaceae	*Spirosoma sp.*
acsa_bin.24.orig	ERS27163285	85.06	0.446	82.83	0.556	6220	4.864311	Spirosomaceae	*Spirosoma sp.*
abba_bin.31.strict	ERS27163204	79.1	3.025	63.975	0.491	8700	5.153156	Spirosomaceae	*Spirosoma sp.*
acsa_bin.12.orig	ERS27163276	77.5	1.636	69.32	0.488	4192	5.251929	Spirosomaceae	*Spirosoma sp.*
abba_bin.40.orig	ERS27163214	66.16	1.19	60.21	0.488	3182	5.01842	Spirosomaceae	*Spirosoma sp.*
all_bin.95	ERS27163371	63.79	3.448	46.55	0.557	8932	4.482887	Spirosomaceae	*Spirosoma sp.*
thoc_bin.106.strict	ERS27163494	60.14	0.744	56.42	0.552	3093	3.125442	Spirosomaceae	*Spirosoma sp.*
all_bin.113	ERS27163312	59.8	5.902	30.29	0.489	7929	3.080929	Spirosomaceae	*Spirosoma sp.*
all_bin.47	ERS27163339	53.6	0	53.6	0.495	10629	4.422678	Spirosomaceae	*Spirosoma sp.*
thoc_bin.108.strict	ERS27163496	53.49	2.998	38.5	0.48	2588	4.339126	Spirosomaceae	*Spirosoma sp.*

all_bin.110	ERS27163310	55.4	0.98	50.5	0.337	3510	0.959613	Weeksellaceae	*Chryseobacterium sp.*
fagr_bin.29.strict	ERS27163447	93.49	0.793	89.525	0.417	37747	3.672803	Sphingobacteriaceae	*Mucilaginibacter sp.*
abba_bin.75.strict	ERS27163249	90.37	3.174	74.5	0.428	10251	3.116422	Sphingobacteriaceae	*Mucilaginibacter sp.*
abba_bin.123.strict	ERS27163186	72.15	0.714	68.58	0.449	4843	2.665611	Sphingobacteriaceae	*Mucilaginibacter sp.*
thoc_bin.64.strict	ERS27163552	60.19	6.386	28.26	0.447	2522	2.612207	Sphingobacteriaceae	*Mucilaginibacter sp.*
coco_bin.46.orig	ERS27163414	87.69	2.96	72.89	0.414	11434	4.463301	Sphingobacteriaceae	*Mucilaginibacter_A sp.*
fagr_bin.18.orig	ERS27163437	79.57	1.071	74.215	0.4	16536	3.628469	Sphingobacteriaceae	*Mucilaginibacter_A sp.*
abba_bin.121.strict	ERS27163184	70.67	0.603	67.655	0.384	6012	3.893778	Sphingobacteriaceae	*Mucilaginibacter_A sp.*
fagr_bin.37.strict	ERS27163454	60.44	1.825	51.315	0.398	5716	3.613472	Sphingobacteriaceae	*Mucilaginibacter_A sp.*
fagr_bin.20.strict	ERS27163440	52.31	2.634	39.14	0.373	4212	4.696698	Sphingobacteriaceae	*Mucilaginibacter_A sp.*
Bdellovibrionota									
all_bin.98	ERS27163374	68.86	9.002	23.85	0.536	4817	3.638326	JAMPXM01	*SXRI01 sp.*
fagr_bin.46.strict	ERS27163463	94.09	0.074	93.72	0.518	44431	4.919633	JAMPXM01	*SXSP01 sp.*
acsa_bin.10.strict	ERS27163274	72.24	0	72.24	0.512	7148	4.316298	JAMPXM01	*SXSP01 sp.*
coco_bin.18.strict	ERS27163384	65.84	3.794	46.87	0.511	5247	4.515062	JAMPXM01	*SXSP01 sp.*
fagr_bin.28.strict	ERS27163446	54.31	0.892	49.85	0.516	2868	2.725279	JAMPXM01	-
all_bin.118	ERS27163314	53.5	0.892	49.04	0.497	4731	2.68908	JAMPXM01	-
all_bin.88	ERS27163367	92.25	5.115	66.675	0.619	10620	3.439705	UBA6776	*JALHOQ01 sp.*
coco_bin.41.strict	ERS27163409	90.57	2.509	78.025	0.545	17108	3.86021	UBA6776	*JALHOQ01 sp.*
abba_bin.73.strict	ERS27163247	75.9	3.993	55.935	0.541	3095	2.560695	UBA6776	*JALHOQ01 sp.*
fagr_bin.2.orig	ERS27163448	71.92	1.116	66.34	0.535	3332	2.481165	UBA6776	*JALHOQ01 sp.*
all_bin.76	ERS27163358	50.87	3.508	33.33	0.58	3939	2.115233	UBA6776	*JALHOQ01 sp.*
all_bin.58	ERS27163346	85.91	4.419	63.815	0.432	7613	2.323669	UBA6776	-
thoc_bin.68.orig	ERS27163555	79.97	1.116	74.39	0.432	3834	2.144531	UBA6776	-
all_bin.71	ERS27163354	72.26	6.14	41.56	0.545	6004	2.331569	UBA6776	-
all_bin.107	ERS27163307	76.49	1.754	67.72	0.547	6716	2.95692	CAIPTA01	*JAKFYM01 sp.*
coco_bin.44.orig	ERS27163412	60.15	0	60.15	0.541	2958	2.340995	CAIPTA01	*JAKFYM01 sp.*
all_bin.104	ERS27163305	58.77	3.508	41.23	0.543	4101	1.832466	CAIPTA01	*JAKFYM01 sp.*
Chlamydiota									
all_bin.37	ERS27163331	86.99	0.675	83.615	0.365	53413	1.553473	FEN-1388	-
all_bin.67	ERS27163351	98.64	2.027	88.505	0.427	94882	2.0951	JABDDJ01	*JABDDJ01 sp.*

thoc_bin.1.strict	ERS27163506	68.85	8.538	26.16	0.325	3234	2.1824	Parachlamydiaceae	*JABDDC01 sp.*
coco_bin.12.orig	ERS27163378	57.16	0.039	56.965	0.391	2821	0.895353	Simkaniaceae	*Neptunochlamydia sp.*
Deinococcota									
thoc_bin.38.orig	ERS27163524	82.19	3.235	66.015	0.682	5191	3.792329	Deinococcaceae	*Deinococcus sp.*
all_bin.26	ERS27163325	67.24	6.551	34.485	0.657	4558	3.684512	Deinococcaceae	*Deinococcus sp.*
thoc_bin.51.orig	ERS27163539	50.31	1.724	41.69	0.669	2960	2.862612	Deinococcaceae	*Deinococcus sp.*
Dependentiae									
abba_bin.32.strict	ERS27163205	81.39	0	81.39	0.352	56103	0.860196	Dependentiae bacterium	-
thoc_bin.89.strict	ERS27163576	80.89	0	80.89	0.349	780614	0.803736	Dependentiae bacterium	-
all_bin.49	ERS27163340	76	0	76	0.35	45180	0.838439	Dependentiae bacterium	-
Myxococcota									
coco_bin.31.orig	ERS27163398	79.89	3.919	60.295	0.701	3662	7.311075	Myxococcaceae	-
thoc_bin.87.orig	ERS27163574	72.57	4.562	49.76	0.701	3752	7.006316	Myxococcaceae	-
all_bin.44	ERS27163337	54.88	8.771	11.025	0.702	4103	7.233065	Myxococcaceae	-
abba_bin.82.orig	ERS27163257	80.51	5.483	53.095	0.668	5963	8.045191	JAEUJZ01	*JAGNNG01 sp.*
thoc_bin.84.orig	ERS27163572	53.27	1.497	45.785	0.685	2735	3.805473	Polyangiaceae	*JAAFHJ01 sp.*
abba_bin.4.strict	ERS27163224	52.38	0.45	50.13	0.686	2571	3.341598	Polyangiaceae	*JAAFHJ01 sp.*
thoc_bin.3.orig	ERS27163526	86.52	2.473	74.155	0.659	6685	6.35157	JALHOP01	-
abba_bin.58.orig	ERS27163232	65.92	1.741	57.215	0.604	3280	4.559103	JALHOP01	-
all_bin.115	ERS27163313	85.23	7.44	48.03	0.602	14422	7.181274	SYDY01	-
abba_bin.64.strict	ERS27163238	68.06	0.798	64.07	0.649	9381	2.189148	SYDY01	-
thoc_bin.101.strict	ERS27163489	66.59	3.294	50.12	0.61	5174	5.126744	SYDY01	-
all_bin.23	ERS27163322	59.49	1.935	49.815	0.57	6507	1.860845	SYDY01	-
Patescibacteria									
thoc_bin.71.orig	ERS27163559	65.41	1.028	60.27	0.489	4378	1.261149	UBA4665	*JACMPA01 sp.*
fagr_bin.19.strict	ERS27163438	53.41	0.522	50.8	0.538	4594	0.603607	Patescibacteria bacterium	-
Planctomycetota									
abba_bin.39.orig	ERS27163212	84.8	1.299	78.305	0.662	6150	6.894796	Isosphaeraceae	*JAJYCH01 sp.*
Pseudomonadota									
abba_bin.14.orig	ERS27163187	74.95	3.332	58.29	0.682	6133	2.178393	Acetobacteraceae	*Acidocella sp.*
all_bin.10	ERS27163309	74.72	3.731	56.065	0.68	8696	2.330971	Acetobacteraceae	*Acidocella sp.*

coco_bin.16.orig	ERS27163382	77.94	1.786	69.01	0.57	4019	2.64327	Acetobacteraceae	*Asaia sp.*
thoc_bin.37.orig	ERS27163523	66.98	1.616	58.9	0.711	2954	3.563298	Acetobacteraceae	*Belnapia sp.*
abba_bin.50.orig	ERS27163225	71.55	0	71.55	0.673	10389	3.357027	Acetobacteraceae	*BOG-908 sp.*
thoc_bin.60.orig	ERS27163549	75.53	1.99	65.58	0.654	7956	5.888739	Acetobacteraceae	*CAHJXG01 sp.*
abba_bin.41.orig	ERS27163215	72.18	1.757	63.395	0.697	5306	4.690475	Acetobacteraceae	*CAHJXG01 sp.*
coco_bin.20.orig	ERS27163387	71.99	2.487	59.555	0.677	13958	4.486642	Acetobacteraceae	*CAHJXG01 sp.*
coco_bin.59.orig	ERS27163423	70.57	1.243	64.355	0.718	6056	3.704528	Acetobacteraceae	*CAHJXG01 sp.*
abba_bin.101.orig	ERS27163164	64.56	9.635	16.385	0.706	5196	4.012778	Acetobacteraceae	*CAHJXG01 sp.*
all_bin.80	ERS27163361	62.51	6.896	28.03	0.669	14564	3.898467	Acetobacteraceae	*CAHJXG01 sp.*
coco_bin.30.orig	ERS27163397	57.75	0	57.75	0.708	6320	3.906163	Acetobacteraceae	*CAHJXG01 sp.*
abba_bin.7.orig	ERS27163254	55.79	0.156	55.01	0.681	7332	3.545189	Acetobacteraceae	*CAHJXG01 sp.*
thoc_bin.47.orig	ERS27163534	53.73	0	53.73	0.673	14091	3.789064	Acetobacteraceae	*CAHJXG01 sp.*
fagr_bin.26.orig	ERS27163445	52.68	3.275	36.305	0.647	3411	6.362061	Acetobacteraceae	*CAHJXG01 sp.*
acsa_bin.3.orig	ERS27163296	52.59	1.907	43.055	0.707	6336	2.964046	Acetobacteraceae	*CAHJXG01 sp.*
abba_bin.3.orig	ERS27163213	51.57	2.985	36.645	0.666	11422	3.188383	Acetobacteraceae	*CAHJXG01 sp.*
fagr_bin.44.orig	ERS27163461	50.05	1.724	41.43	0.723	3441	3.363245	Acetobacteraceae	*CAHJXG01 sp.*
abba_bin.68.orig	ERS27163242	90.91	1.492	83.45	0.676	11854	3.758465	Acetobacteraceae	*JAFKFI01 sp.*
abba_bin.62.orig	ERS27163237	94.53	1.658	86.24	0.698	19105	3.341156	Acetobacteraceae	*JAJXVA01 sp.*
abba_bin.117.orig	ERS27163179	80.2	3.383	63.285	0.677	6203	2.951263	Acetobacteraceae	*JAJXVA01 sp.*
thoc_bin.67.orig	ERS27163554	86.8	1.523	79.185	0.659	8857	4.717598	Acetobacteraceae	*Lichenicoccus sp.*
all_bin.87	ERS27163366	54.85	3.39	37.9	0.656	11784	3.675594	Acetobacteraceae	*Lichenicoccus sp.*
all_bin.82	ERS27163363	51.2	1.724	42.58	0.69	5976	3.288599	Acetobacteraceae	*Lichenicoccus sp.*
all_bin.43	ERS27163336	62.24	7.839	23.045	0.7	4672	2.864691	Acetobacteraceae	*Lichenicola sp.*
abba_bin.78.strict	ERS27163252	55.17	1.724	46.55	0.688	10308	4.457879	Acetobacteraceae	*Lichenicola sp.*
abba_bin.9.strict	ERS27163273	64.81	2.411	52.755	0.711	6377	3.197394	Acetobacteraceae	*LMUY01 sp.*
abba_bin.79.orig	ERS27163253	57.18	0	57.18	0.7	4149	3.03666	Acetobacteraceae	*LMUY01 sp.*
abba_bin.52.orig	ERS27163227	50.56	3.396	33.58	0.684	4652	3.916429	Acetobacteraceae	*LMUY01 sp.*
abba_bin.76.orig	ERS27163250	95.4	1.16	89.6	0.61	18376	5.73149	Acetobacteraceae	*Rhodopila sp.*
abba_bin.37.orig	ERS27163210	93.58	3.109	78.035	0.627	11420	6.004671	Acetobacteraceae	*Rhodopila sp.*
abba_bin.49.orig	ERS27163223	91.96	0.414	89.89	0.599	23816	5.480659	Acetobacteraceae	*Rhodopila sp.*
all_bin.102	ERS27163304	86.4	7.504	48.88	0.633	12600	5.238691	Acetobacteraceae	*Rhodopila sp.*

abba_bin.113.strict	ERS27163175	80.84	2.238	69.65	0.597	8802	4.398897	Acetobacteraceae	*Rhodopila sp.*
abba_bin.53.orig	ERS27163228	64.31	4.394	42.34	0.644	4171	4.783688	Acetobacteraceae	*Rhodopila sp.*
all_bin.97	ERS27163373	57.86	3.233	41.695	0.652	22663	4.343509	Acetobacteraceae	*Rhodopila sp.*
abba_bin.114.strict	ERS27163176	56.55	3.034	41.38	0.574	3528	3.620446	Acetobacteraceae	*Rhodopila sp.*
thoc_bin.82.orig	ERS27163570	55.72	3.229	39.575	0.601	3060	3.904642	Acetobacteraceae	*Rhodopila sp.*
coco_bin.36.orig	ERS27163403	74.95	1.824	65.83	0.667	3355	2.644519	Acetobacteraceae	*Tanticharoenia sp.*
fagr_bin.5.orig	ERS27163477	50.91	6.896	16.43	0.719	3146	2.749694	Acetobacteraceae	-
fagr_bin.58.strict	ERS27163475	80.91	0	80.91	0.423	113991	1.490724	Caedimonadaceae	-
fagr_bin.6.orig	ERS27163484	78.52	0.434	76.35	0.328	15620	1.499873	Caedimonadaceae	-
all_bin.99	ERS27163375	65.21	8.442	23	0.395	6283	1.201516	Caedimonadaceae	-
abba_bin.91.strict	ERS27163264	59.3	0.826	55.17	0.424	5471	1.207311	Caedimonadaceae	-
acsa_bin.36.strict	ERS27163294	57.21	1.63	49.06	0.375	3852	1.382606	Caedimonadaceae	-
acsa_bin.6.permissive	ERS27163299	62.75	0.097	62.265	0.403	4483	0.895296	UBA9215	-
all_bin.16	ERS27163318	59.1	1.075	53.725	0.4	6108	0.842865	UBA9215	-
thoc_bin.74.orig	ERS27163561	69.49	1.785	60.565	0.719	5823	2.575924	Caulobacteraceae	*CAHJWH01 sp.*
all_bin.59	ERS27163347	52.58	1.724	43.96	0.724	7802	1.674472	Caulobacteraceae	*CAHJWH01 sp.*
all_bin.83	ERS27163364	77.51	1.646	69.28	0.655	11792	2.449174	Caulobacteraceae	*Palsa-881 sp.*
thoc_bin.2.orig	ERS27163515	65.67	3.144	49.95	0.716	4523	2.583633	Caulobacteraceae	*PMMR1 sp.*
thoc_bin.58.orig	ERS27163546	52.16	9.31	5.61	0.704	2413	5.690981	Caulobacteraceae	*PMMR1 sp.*
all_bin.89	ERS27163368	90.32	0	90.32	0.356	11831	1.489312	CAIULA01	-
thoc_bin.102.orig	ERS27163490	78.1	1.044	72.88	0.704	9171	3.32354	Beijerinckiaceae	*Enterovirga sp.*
abba_bin.25.orig	ERS27163197	91.68	1.458	84.39	0.625	8739	3.194673	Beijerinckiaceae	*JAJXWB01 sp.*
thoc_bin.79.orig	ERS27163566	85.49	2.318	73.9	0.624	5907	2.965544	Beijerinckiaceae	*JAJXWB01 sp.*
abba_bin.105.strict	ERS27163168	60.5	6.035	30.325	0.649	2759	2.972097	Beijerinckiaceae	*JAJXWB01 sp.*
all_bin.63	ERS27163349	60.32	3.291	43.865	0.626	3699	2.224184	Beijerinckiaceae	*JAJXWB01 sp.*
abba_bin.103.orig	ERS27163166	52.32	6.896	17.84	0.657	2600	2.443038	Beijerinckiaceae	*JAJXWB01 sp.*
abba_bin.70.orig	ERS27163245	82.19	2.037	72.005	0.646	10553	4.397224	Beijerinckiaceae	*Lichenihabitans sp.*
coco_bin.9.orig	ERS27163429	81.32	2.351	69.565	0.66	10419	4.036797	Beijerinckiaceae	*Lichenihabitans sp.*
abba_bin.112.orig	ERS27163174	79.86	8.408	37.82	0.662	10774	4.806598	Beijerinckiaceae	*Lichenihabitans sp.*
abba_bin.35.orig	ERS27163208	79.37	5.245	53.145	0.697	6166	4.569629	Beijerinckiaceae	*Lichenihabitans sp.*
abba_bin.120.orig	ERS27163183	78.79	2.15	68.04	0.666	11702	3.844366	Beijerinckiaceae	*Lichenihabitans sp.*

thoc_bin.99.orig	ERS27163585	78.76	2.542	66.05	0.658	9030	4.512106	Beijerinckiaceae	*Lichenihabitans sp.*
fagr_bin.54.orig	ERS27163471	69.71	2.298	58.22	0.661	11600	3.401858	Beijerinckiaceae	*Lichenihabitans sp.*
abba_bin.60.strict	ERS27163235	68.67	1.724	60.05	0.701	12295	5.26731	Beijerinckiaceae	*Lichenihabitans sp.*
abba_bin.66.orig	ERS27163240	66.7	1.316	60.12	0.651	4740	3.759278	Beijerinckiaceae	*Lichenihabitans sp.*
abba_bin.36.orig	ERS27163209	66.65	6.034	36.48	0.682	6545	3.15816	Beijerinckiaceae	*Lichenihabitans sp.*
acsa_bin.11.orig	ERS27163275	66.29	1.724	57.67	0.653	6250	3.00825	Beijerinckiaceae	*Lichenihabitans sp.*
fagr_bin.8.orig	ERS27163486	58.59	1.793	49.625	0.711	3939	3.094881	Beijerinckiaceae	*Lichenihabitans sp.*
all_bin.68	ERS27163352	56.89	6.034	26.72	0.703	13073	3.241374	Beijerinckiaceae	*Lichenihabitans sp.*
thoc_bin.29.orig	ERS27163514	55.17	6.556	22.39	0.637	8023	3.555302	Beijerinckiaceae	*Lichenihabitans sp.*
abba_bin.29.orig	ERS27163201	54.26	3.448	37.02	0.638	3842	4.742143	Beijerinckiaceae	*Lichenihabitans sp.*
abba_bin.23.orig	ERS27163195	53.52	4.31	31.97	0.694	4709	3.645119	Beijerinckiaceae	*Lichenihabitans sp.*
thoc_bin.46.orig	ERS27163533	53.05	0	53.05	0.673	4404	3.267498	Beijerinckiaceae	*Lichenihabitans sp.*
abba_bin.88.orig	ERS27163261	79.88	0.909	75.335	0.716	7648	4.070851	Beijerinckiaceae	*Methylobacterium sp.*
all_bin.20	ERS27163320	59.05	8.317	17.465	0.693	4256	2.807154	Beijerinckiaceae	*Methylobacterium sp.*
coco_bin.25.orig	ERS27163392	56.9	4.085	36.475	0.714	3916	3.444893	Beijerinckiaceae	*Methylobacterium sp.*
coco_bin.40.orig	ERS27163408	56.2	1.724	47.58	0.696	3782	3.013607	Beijerinckiaceae	*Methylobacterium sp.*
abba_bin.87.orig	ERS27163260	51.89	0.862	47.58	0.729	3758	3.049443	Beijerinckiaceae	*Methylobacterium sp.*
fagr_bin.53.orig	ERS27163470	51.14	2.413	39.075	0.714	3771	2.92056	Beijerinckiaceae	*Methylobacterium sp.*
fagr_bin.34.orig	ERS27163451	58.75	0.877	54.365	0.712	4314	2.815876	Beijerinckiaceae	*Methylobacterium sp008039875*
coco_bin.39.orig	ERS27163406	53.73	1.253	47.465	0.72	3472	2.591356	Beijerinckiaceae	*Methylobacterium sp008039875*
acsa_bin.28.orig	ERS27163289	51.68	1.802	42.67	0.713	5321	2.608492	Beijerinckiaceae	*Methylobacterium sp008039875*
thoc_bin.88.orig	ERS27163575	79.93	0.975	75.055	0.682	13103	5.436576	Beijerinckiaceae	*Methylobacterium sp022829605*
abba_bin.20.orig	ERS27163194	58.07	0	58.07	0.689	5319	4.217813	Beijerinckiaceae	*Methylobacterium sp022829605*
coco_bin.21.orig	ERS27163388	50.33	6.491	17.875	0.694	3235	3.726788	Beijerinckiaceae	*Methylobacterium sp022829605*
coco_bin.48.orig	ERS27163416	78.94	1.201	72.935	0.681	11179	3.043255	Beijerinckiaceae	*RH-AL1 sp.*
thoc_bin.73.orig	ERS27163560	78.86	1.913	69.295	0.684	7458	2.886432	Beijerinckiaceae	*RH-AL1 sp.*
thoc_bin.75.orig	ERS27163562	65.73	0.888	61.29	0.658	5660	2.504697	Beijerinckiaceae	*RH-AL1 sp.*
abba_bin.43.orig	ERS27163217	64.73	0.862	60.42	0.658	6395	2.464629	Beijerinckiaceae	*RH-AL1 sp.*
fagr_bin.48.orig	ERS27163464	64.59	2.542	51.88	0.684	4152	3.126019	Beijerinckiaceae	*RH-AL1 sp.*
coco_bin.29.orig	ERS27163395	62.77	2.507	50.235	0.689	3910	3.051752	Beijerinckiaceae	*RH-AL1 sp.*
fagr_bin.15.orig	ERS27163434	60.6	6.687	27.165	0.643	2684	2.828606	Beijerinckiaceae	*RH-AL1 sp.*

abba_bin.85.orig	ERS27163259	59.9	1.036	54.72	0.678	4243	2.388927	Beijerinckiaceae	*RH-AL1 sp.*
thoc_bin.80.orig	ERS27163568	59.84	0.783	55.925	0.678	4780	2.363868	Beijerinckiaceae	*RH-AL1 sp.*
coco_bin.1.orig	ERS27163386	59.24	1.776	50.36	0.654	4739	2.282496	Beijerinckiaceae	*RH-AL1 sp.*
acsa_bin.30.orig	ERS27163291	57.79	3.014	42.72	0.651	5580	2.145871	Beijerinckiaceae	*RH-AL1 sp.*
fagr_bin.23.strict	ERS27163443	57.75	0	57.75	0.659	6368	2.607785	Beijerinckiaceae	*RH-AL1 sp.*
all_bin.108	ERS27163308	51.01	5.52	23.41	0.693	4355	2.443934	Beijerinckiaceae	*RH-AL1 sp.*
thoc_bin.45.orig	ERS27163532	86.58	0.626	83.45	0.653	15990	3.713847	Beijerinckiaceae	-
all_bin.60	ERS27163348	77.07	3.731	58.415	0.651	15028	3.534844	Beijerinckiaceae	-
coco_bin.11.orig	ERS27163377	62.17	9.482	14.76	0.661	2617	4.866406	Beijerinckiaceae	-
coco_bin.2.orig	ERS27163396	65.61	2.93	50.96	0.673	3298	3.022559	Rhizobiaceae	*Aureimonas_A sp.*
acsa_bin.29.orig	ERS27163290	98.9	0.549	96.155	0.274	123289	1.576301	Arcanobacteraceae	-
fagr_bin.70.orig	ERS27163485	92.19	4.439	69.995	0.271	9339	1.384205	Arcanobacteraceae	-
acsa_bin.19.orig	ERS27163280	70.61	4.851	46.355	0.279	3761	1.118925	Arcanobacteraceae	-
fagr_bin.61.orig	ERS27163478	93.36	1.098	87.87	0.314	15409	1.094655	Midichloriaceae	*JAJTHY01 sp.*
thoc_bin.53.permissive	ERS27163541	100	0.549	97.255	0.327	184321	1.403527	Midichloriaceae	-
all_bin.81	ERS27163362	94.5	1.098	89.01	0.306	34930	1.396546	Midichloriaceae	-
acsa_bin.34.orig	ERS27163293	76.42	1.938	66.73	0.326	3961	0.989158	Rickettsiaceae	*GCF-002259525 sp020410785*
all_bin.78	ERS27163359	55.47	3.439	38.275	0.327	4185	1.037929	Rickettsiaceae	*GCF-002259525 sp020410785*
fagr_bin.14.orig	ERS27163433	84.3	1.846	75.07	0.326	4583	1.16528	Rickettsiaceae	*GCF-002259525 sp020881075*
thoc_bin.16.orig	ERS27163503	99.52	0.868	95.18	0.316	376014	1.429478	Rickettsiaceae	*JAKONF01 sp.*
abba_bin.1.permissive	ERS27163193	97.94	1.754	89.17	0.312	38593	1.624397	Rickettsiaceae	*JAKONF01 sp.*
fagr_bin.16.orig	ERS27163435	96.91	1.421	89.805	0.316	9918	1.431792	Rickettsiaceae	*JAKONF01 sp.*
thoc_bin.8.strict	ERS27163577	94.94	0.552	92.18	0.337	51957	1.447786	Rickettsiaceae	*JAKONF01 sp.*
abba_bin.28.orig	ERS27163200	89.57	2.613	76.505	0.324	7083	1.401145	Rickettsiaceae	*JAKONF01 sp.*
coco_bin.28.strict	ERS27163394	89.35	3.728	70.71	0.312	8210	1.32582	Rickettsiaceae	*JAKONF01 sp.*
thoc_bin.83.permissive	ERS27163571	89.25	1.859	79.955	0.318	18993	1.286896	Rickettsiaceae	*JAKONF01 sp.*
coco_bin.32.orig	ERS27163399	80.67	3.87	61.32	0.315	4500	1.175218	Rickettsiaceae	*JAKONF01 sp.*
coco_bin.45.strict	ERS27163413	80.14	9.274	33.77	0.313	5856	1.514314	Rickettsiaceae	*JAKONF01 sp.*
coco_bin.38.orig	ERS27163405	99.28	2.606	86.25	0.316	78490	1.510388	Rickettsiaceae	-
acsa_bin.13.orig	ERS27163277	98.1	1.421	90.995	0.313	146899	1.262903	Rickettsiaceae	-
thoc_bin.18.strict	ERS27163504	95.73	0.947	90.995	0.315	29749	1.32447	Rickettsiaceae	-

thoc_bin.69.orig	ERS27163556	93.83	0.71	90.28	0.318	24278	1.14268	Rickettsiaceae	-
fagr_bin.49.strict	ERS27163465	91.23	1.759	82.435	0.301	13817	1.411945	Rickettsiaceae	-
thoc_bin.59.orig	ERS27163547	67.79	2.132	57.13	0.304	4066	0.935058	Rickettsiaceae	-
abba_bin.8.orig	ERS27163262	63.91	1.468	56.57	0.704	3787	2.103108	Sphingomonadaceae	*CAHJWT01 sp.*
abba_bin.18.strict	ERS27163191	69.7	2.834	55.53	0.644	6669	3.120326	Sphingomonadaceae	*Novosphingobium sp.*
abba_bin.74.orig	ERS27163248	65.58	2.056	55.3	0.655	5296	3.398932	Sphingomonadaceae	*Novosphingobium sp.*
thoc_bin.52.orig	ERS27163540	75.71	2.872	61.35	0.65	7031	3.272895	Sphingomonadaceae	*Sphingomonas sp.*
abba_bin.51.orig	ERS27163226	63.77	1.56	55.97	0.696	4015	2.16851	Sphingomonadaceae	*Sphingomonas sp.*
fagr_bin.3.orig	ERS27163457	60.34	2.586	47.41	0.689	3927	2.179126	Sphingomonadaceae	*Sphingomonas sp.*
coco_bin.33.orig	ERS27163400	59.8	0.35	58.05	0.692	8903	2.512683	Sphingomonadaceae	*Sphingomonas sp.*
coco_bin.22.orig	ERS27163389	58.31	2.03	48.16	0.691	5533	2.005285	Sphingomonadaceae	*Sphingomonas sp.*
all_bin.25	ERS27163324	57.15	2.58	44.25	0.65	8504	2.355273	Sphingomonadaceae	*Sphingomonas sp.*
thoc_bin.103.orig	ERS27163491	52.69	1.754	43.92	0.704	3111	1.95799	Sphingomonadaceae	*Sphingomonas sp.*
acsa_bin.21.orig	ERS27163282	51.48	1.754	42.71	0.69	6949	2.356452	Sphingomonadaceae	*Sphingomonas sp.*
coco_bin.35.orig	ERS27163402	50.58	0.877	46.195	0.71	3980	2.477927	Sphingomonadaceae	*Sphingomonas sp.*
abba_bin.110.strict	ERS27163172	62.02	0	62.02	0.648	5462	2.631783	Sphingomonadaceae	*Sphingomonas_I sp.*
fagr_bin.57.orig	ERS27163474	52.58	0.862	48.27	0.657	3051	2.094557	Sphingomonadaceae	*Sphingomonas_I sp.*
fagr_bin.42.orig	ERS27163459	51.72	3.448	34.48	0.692	2813	2.04127	Sphingomonadaceae	*Sphingomonas_I sp.*
coco_bin.51.orig	ERS27163419	66.11	1.809	57.065	0.708	5033	2.176124	Sphingomonadaceae	*Sphingomonas_N sp.*
fagr_bin.55.orig	ERS27163472	65.57	1.304	59.05	0.679	5277	2.713301	Sphingomonadaceae	*Sphingomonas_N sp.*
thoc_bin.50.orig	ERS27163538	55.5	5.172	29.64	0.704	3584	2.417803	Sphingomonadaceae	*Sphingomonas_N sp.*
abba_bin.102.orig	ERS27163165	54.13	1.724	45.51	0.703	8266	2.400481	Sphingomonadaceae	*Sphingomonas_N sp.*
coco_bin.37.orig	ERS27163404	52.8	5.172	26.94	0.678	2903	2.737864	Sphingomonadaceae	*Sphingomonas_N sp.*
all_bin.1	ERS27163319	65.58	6.39	33.63	0.61	3547	3.710955	Burkholderiaceae	*Caballeronia sp.*
thoc_bin.35.orig	ERS27163521	87.52	1.424	80.4	0.688	17513	3.900277	Burkholderiaceae	*CAHJXF01 sp.*
thoc_bin.92.orig	ERS27163580	86.01	1.414	78.94	0.71	7309	3.881109	Burkholderiaceae	*CAHJXF01 sp.*
abba_bin.54.orig	ERS27163229	78.75	1.851	69.495	0.712	6061	3.633644	Burkholderiaceae	*CAHJXF01 sp.*
abba_bin.65.orig	ERS27163239	53.79	1.724	45.17	0.689	5595	2.285452	Burkholderiaceae	*CAHJXF01 sp.*
all_bin.38	ERS27163332	57.24	5.172	31.38	0.627	7304	8.437279	Burkholderiaceae	*Telluria sp.*
coco_bin.61.orig	ERS27163426	55.41	6.347	23.675	0.625	2107	3.35567	Burkholderiaceae	*Telluria sp.*
coco_bin.23.orig	ERS27163390	62.57	3.621	44.465	0.691	3089	3.908446	Burkholderiaceae_B	*CAIKUF01 sp.*

all_bin.84	ERS27163365	65.66	7.472	28.3	0.716	3665	3.568011	Burkholderiaceae_B	*CAIMXF01 sp.*
thoc_bin.49.orig	ERS27163536	79.54	2.27	68.19	0.71	6392	3.386635	Burkholderiaceae_B	*LMDS01 sp.*
coco_bin.7.orig	ERS27163428	67.58	0	67.58	0.713	3280	2.755181	Burkholderiaceae_B	*LMDS01 sp.*
abba_bin.46.orig	ERS27163220	59.48	0	59.48	0.708	4409	2.562104	Burkholderiaceae_B	*LMDS01 sp.*
fagr_bin.36.orig	ERS27163453	56.89	0	56.89	0.708	3824	2.417579	Burkholderiaceae_B	*LMDS01 sp.*
all_bin.91	ERS27163369	51.37	1.724	42.75	0.71	6675	2.676287	Burkholderiaceae_B	*LMDS01 sp.*
thoc_bin.25.orig	ERS27163510	61.44	1.09	55.99	0.687	2855	2.492859	Burkholderiaceae_B	*Schlegelella_A sp.*
coco_bin.56.orig	ERS27163421	96.21	1.375	89.335	0.689	15788	4.786202	Burkholderiaceae_B	*Variovorax sp.*
coco_bin.43.orig	ERS27163411	79.19	1.5	71.69	0.699	4133	3.187558	Burkholderiaceae_B	*Xylophilus sp.*
fagr_bin.1.orig	ERS27163439	93.02	0	93.02	0.369	445110	1.527027	Diplorickettsiaceae	*Aquirickettsiella sp.*
coco_bin.24.orig	ERS27163391	100	0	100	0.258	120386	0.644462	Enterobacteriaceae	*Buchnera sp.*
acsa_bin.26.orig	ERS27163287	89.99	2.905	75.465	0.564	5980	4.544816	Enterobacteriaceae	*Erwinia aphidicola*
fagr_bin.62.orig	ERS27163479	100	0.575	97.125	0.538	92697	5.527551	Enterobacteriaceae	*Erwinia billingiae*
thoc_bin.11.strict	ERS27163499	75.82	1.057	70.535	0.549	3823	3.788234	Enterobacteriaceae	*Erwinia billingiae*
abba_bin.108.strict	ERS27163170	97.13	0.107	96.595	0.47	26607	3.490165	Enterobacteriaceae	-
acsa_bin.37.orig	ERS27163295	80.51	0.113	79.945	0.188	6255	0.643804	Enterobacteriaceae	-
all_bin.79	ERS27163360	90.56	0	90.56	0.385	23790	1.277356	JAJYDF01	-
fagr_bin.51.orig	ERS27163468	96.78	1.332	90.12	0.366	49189	2.690976	Legionellaceae	*Legionella_C sp.*
all_bin.55	ERS27163344	82.09	3.313	65.525	0.356	19583	1.960662	Legionellaceae	*Legionella_C sp.*
thoc_bin.15.orig	ERS27163502	96.57	1.166	90.74	0.585	67448	5.25615	Pseudomonadaceae	*Pseudomonas_E sp.*
acsa_bin.7.orig	ERS27163300	89.26	2.246	78.03	0.64	12276	4.801572	Pseudomonadaceae	*Pseudomonas_E sp.*
thoc_bin.43.strict	ERS27163530	87.8	1.564	79.98	0.547	8110	4.393315	Pseudomonadaceae	*Pseudomonas_E sp.*
acsa_bin.23.orig	ERS27163284	61.75	0	61.75	0.591	5010	5.259721	Pseudomonadaceae	*Pseudomonas_E sp.*
acsa_bin.8.orig	ERS27163301	98.56	0.781	94.655	0.607	72272	6.450392	Pseudomonadaceae	*Pseudomonas_E sp003014915*
fagr_bin.66.orig	ERS27163482	50.87	4.385	28.945	0.611	4529	5.072014	Pseudomonadaceae	*Pseudomonas_E sp003014915*
acsa_bin.32.strict	ERS27163292	96.77	0	96.77	0.364	399529	1.466389	Pseudomonadota bacterium	-
thoc_bin.48.orig	ERS27163535	95.69	0.097	95.205	0.385	58978	1.485475	Pseudomonadota bacterium	-
acsa_bin.1.orig	ERS27163281	92.62	0	92.62	0.389	15203	1.412951	Pseudomonadota bacterium	-
acsa_bin.27.strict	ERS27163288	87.99	0.664	84.67	0.402	33314	1.123851	Pseudomonadota bacterium	-
all_bin.39	ERS27163333	83.87	2.329	72.225	0.395	14440	1.398822	Pseudomonadota bacterium	-
fagr_bin.52.strict	ERS27163469	79.52	4.301	58.015	0.386	13655	1.199901	Pseudomonadota bacterium	-

all_bin.33	ERS27163329	77.81	1.075	72.435	0.382	34577	1.178847	Pseudomonadota bacterium	-
acsa_bin.22.permissive	ERS27163283	72.8	0.374	70.93	0.381	4331	1.115408	Pseudomonadota bacterium	-
all_bin.36	ERS27163330	71.55	2.688	58.11	0.406	6384	0.767232	Pseudomonadota bacterium	-
acsa_bin.9.strict	ERS27163302	68.1	0	68.1	0.393	10420	1.070878	Pseudomonadota bacterium	-
acsa_bin.4.orig	ERS27163297	66.27	5.913	36.705	0.385	3538	1.154176	Pseudomonadota bacterium	-
fagr_bin.33.orig	ERS27163450	56.45	0.391	54.495	0.386	3688	1.00249	Pseudomonadota bacterium	-
coco_bin.6.permissive	ERS27163427	95.6	0.064	95.28	0.319	15733	1.828253	Pseudomonadota bacterium	-
acsa_bin.25.orig	ERS27163286	77.07	9.34	30.37	0.387	6108	1.162498	Pseudomonadota bacterium	-
coco_bin.19.strict	ERS27163385	68.8	0.549	66.055	0.377	3567	1.168339	Pseudomonadota bacterium	-
Verrucomicrobiota									
all_bin.11	ERS27163315	63.34	0.675	59.965	0.37	7872	1.049226	Verrucomicrobiota bacterium	-

Table S2: Sequencing information of 25 temperate forest phyllosphere samples from Eastern Canada. Paired-end metagenomic shotgun sequencing was performed using the Illumina NovaSeq 6000 S4 flow cell at the Centre de recherche CHU de Québec (Laval University, Québec, Canada). Reads were trimmed with Trim Galore v0.5.0, and host reads were removed (where possible) using BMTagger v1.1.0.

Sample	Accession (SRA)	Raw reads (M)	Trimmed reads (M)	% Host reads	Filtered reads (M)	bp
ABBAM11	ERS27090401	77.37	57.65	NA	57.65	11759733992
ABBAM12	ERS27090402	87.14	63.82	NA	63.82	13245472888
ABBAM13	ERS27090403	88.78	66.02	NA	66.02	13494609096
ABBAM14	ERS27090404	123.58	92.02	NA	92.02	18783379784
ABBAM15	ERS27090405	81.17	61.52	NA	61.52	12338190208
ACSAM21	ERS27090406	67.86	52.65	0.417853751	52.43	10314272968
ACSAM22	ERS27090407	82.73	62.56	0.495524297	62.25	12574755560
ACSAM23	ERS27090408	54.3	42.83	0.326873687	42.69	8253791064
ACSAM24	ERS27090409	64.83	49.81	0.782975306	49.42	9854472816
ACSAM25	ERS27090410	83.92	61.89	0.678623364	61.47	12756506520
COCOM1	ERS27090411	75.94	56.74	7.772294677	52.33	11543313960
COCOM2	ERS27090412	76.93	57.95	4.245038827	55.49	11693196904
COCOM3	ERS27090413	87.54	64.51	4.665943265	61.5	13306788320
COCOM4	ERS27090414	84.21	61.11	5.776468663	57.58	12800580896
COCOM5	ERS27090415	92.35	66.27	3.531009507	63.93	14037580152
FAGRM16	ERS27090416	93	70.19	2.308021086	68.57	14136590672
FAGRM17	ERS27090417	95.57	70.41	3.167163755	68.18	14526760384
FAGRM18	ERS27090418	105.35	77.56	3.687467767	74.7	16013212616
FAGRM19	ERS27090419	90.25	66.19	1.525910258	65.18	13717661800
FAGRM20	ERS27090420	72.91	54.59	1.70360872	53.66	11081924040
THOCM6	ERS27090421	86.91	64.09	NA	64.09	12990080056
THOCM7	ERS27090422	85.46	61.97	NA	61.97	13472925688
THOCM8	ERS27090423	88.64	64.28	NA	64.28	12796974544
THOCM9	ERS27090424	84.19	61.92	NA	61.92	14604719208
THOCM10	ERS27090425	96.08	68.37	NA	68.37	13210305864

SUPPLEMENTARY FIGURES

Figure S1: Heatmap showing the count, average completeness, and average contamination of all MAGs recovered from each of the 6 co-assemblies (before dereplication), grouped by family. Completeness and contamination were measured using CheckM v1.2.1, taxonomy was assigned with GTDB-Tk v2.3.2 using the GTDB v08 release R214. ABBA = *Abies balsamea* (n=121, from samples ABBAM11-15); THOC = *Thuya occidentalis* (n=107, from samples THOCM6-10); ACSA = *Acer saccharum* (n=37, from samples ACSAM21-25); FAGR = *Fagus grandifolia* (n=68, from samples FAGRM16-20); COCO = *Corylus cornuta* (n=60, from samples COCOM1-5); ALL = all host species (n=120, from all samples).

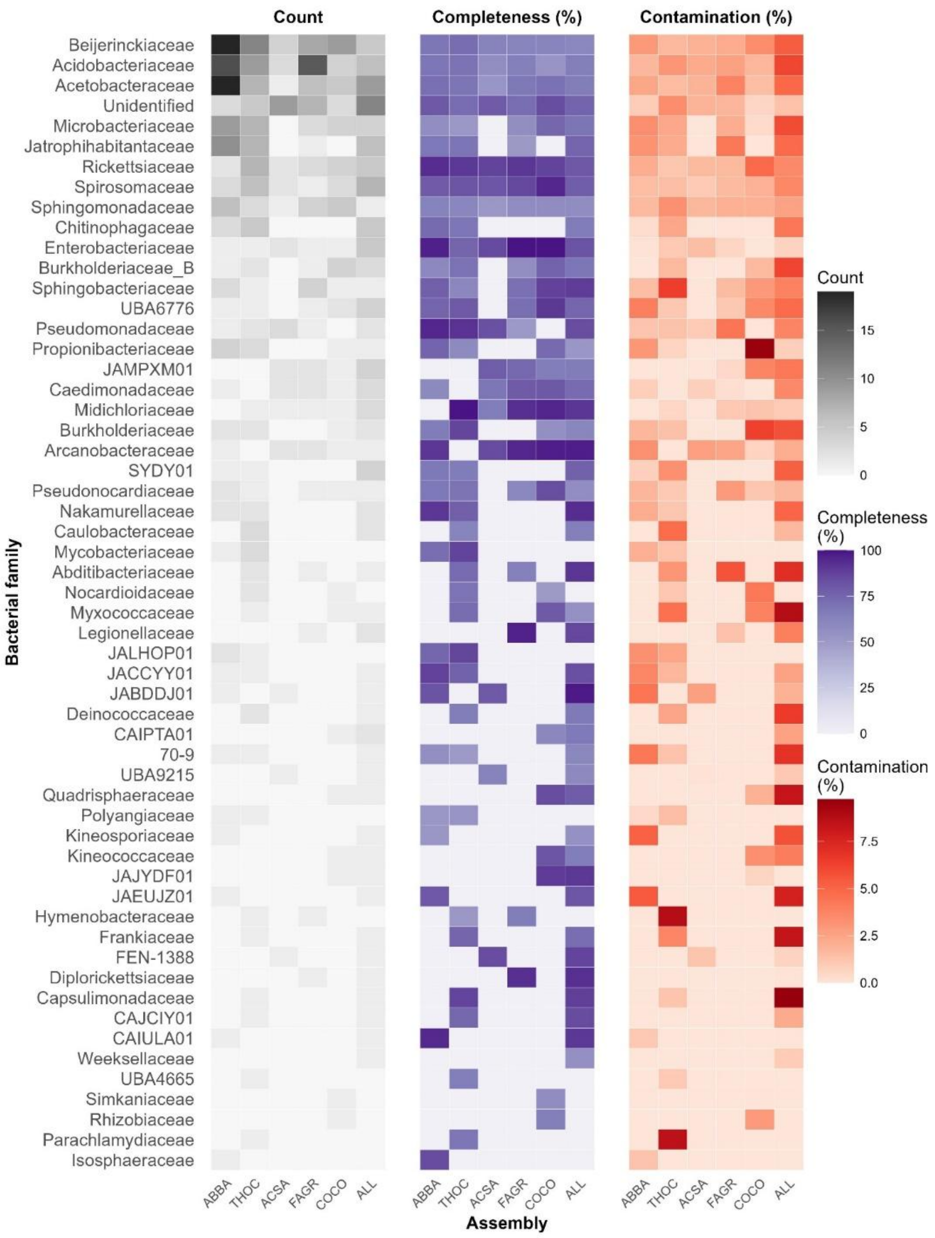

Figure S2: Overlayed violin and boxplot of contamination of all MAGs recovered from each of the 6 co-assemblies (before dereplication), measured using CheckM v1.2.1. ABBA = *Abies balsamea* (samples ABBAM11-15); THOC = *Thuya occidentalis* (samples THOCM6-10); ACSA = *Acer saccharum* (samples ACSAM21-25); FAGR = *Fagus grandifolia* (samples FAGRM16-20); COCO = *Corylus cornuta* (samples COCOM1-5); ALL = all host species combined (all samples). Contamination was significantly higher in ALL than in each of the host-species specific assemblies (MANOVA: Pillais' Trace = 0.12, $F_{(10, 1014)} = 6.28$, $p < 0.001$; ANOVA: $F_{(5, 507)} = 11.21$, $p < 0.001$).

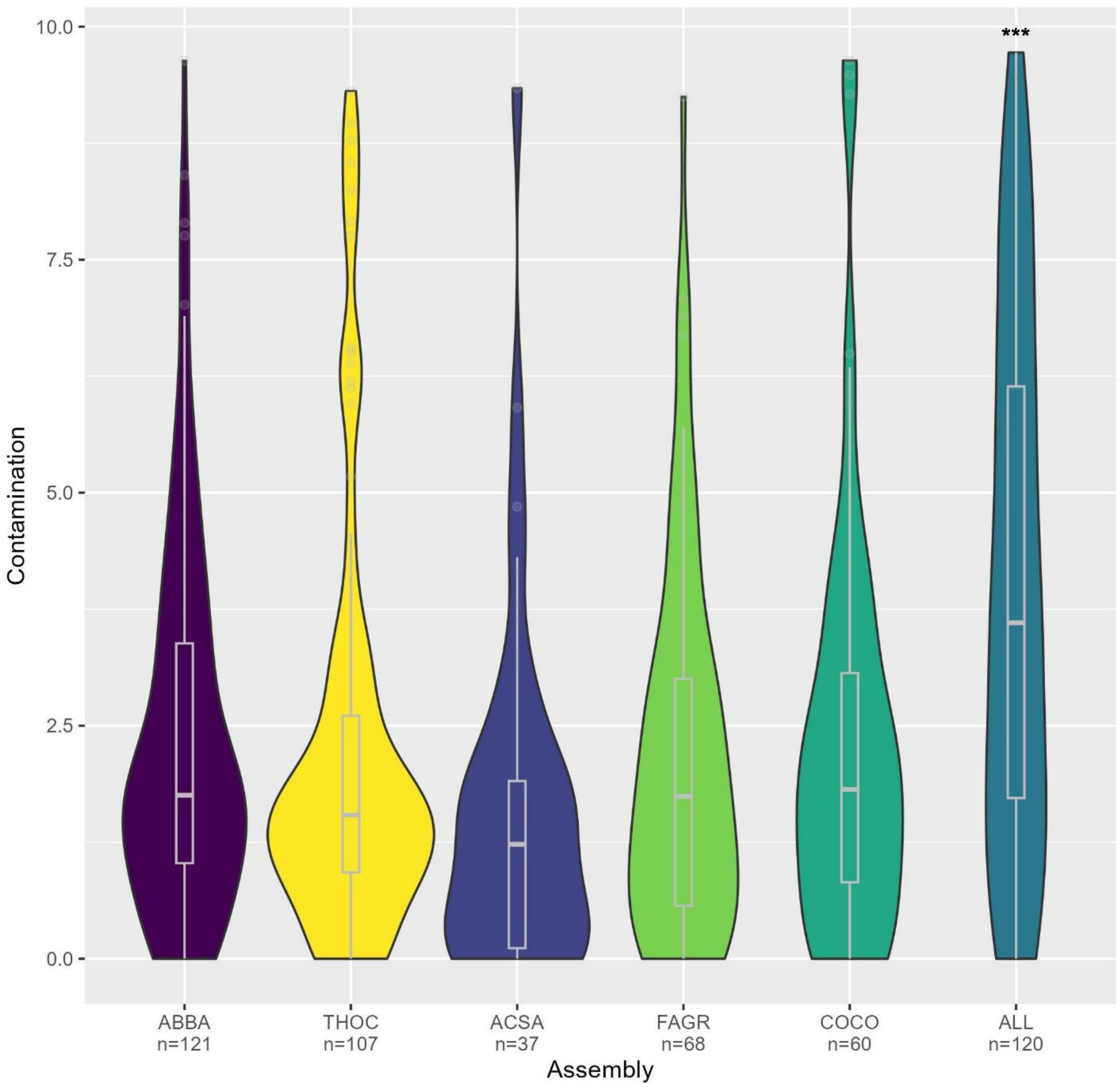

Figure S3: Barplots showing the covered fraction and coverage depth (mean + SD) of the final MAGs (after dereplication), grouped by the co-assembled contigs they were binned from, when each of the concatenated read files were mapped back onto them using CoverM v0.7.0. Each box shows the results for the associated concatenated read file. ABBA = *Abies balsamea* (samples ABBAM11-15); THOC = *Thuya occidentalis* (samples THOCM6-10); ACSA = *Acer saccharum* (samples ACSAM21-25); FAGR = *Fagus grandifolia* (samples FAGRM16-20); COCO = *Corylus cornuta* (samples COCOM1-5); ALL = all host species combined (all samples).

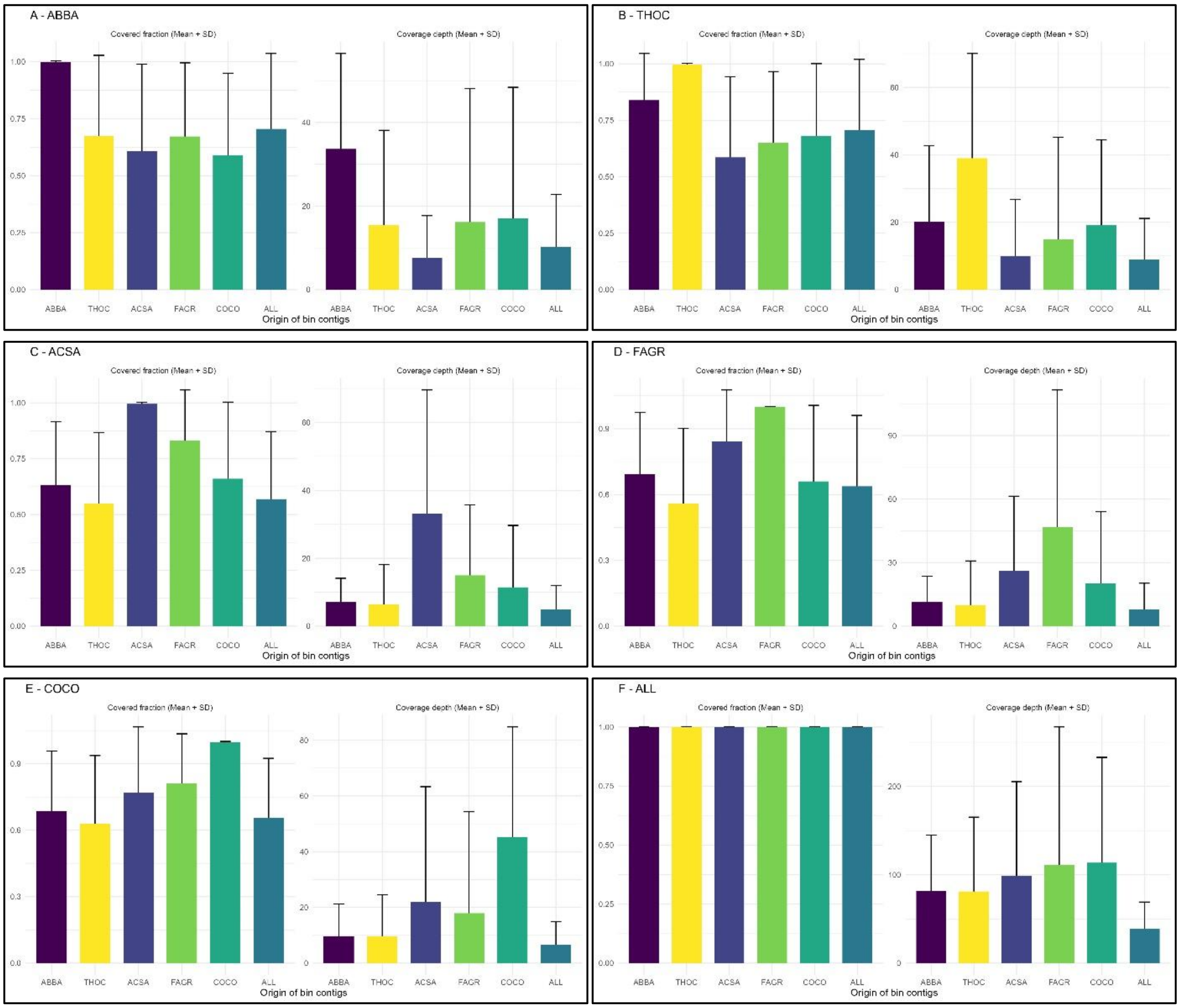

Supplement: Supplementary Material 1. [file acmi-8-01189-s001.pdf]
